# High-throughput sequencing of bronchoalveolar lavage fluid confirms pulmonary paragonimiasis: A case report

**DOI:** 10.1097/MD.0000000000045261

**Published:** 2025-10-24

**Authors:** Junzhang Qin, Weilu Zhu, Zhen Xiong

**Affiliations:** a The Department of Respiratory and Critical Care Medicine, The 924th Hospital of the Joint Logistics Support Force of the Chinese People’s Liberation Army, Guilin City, Guangxi Zhuang Autonomous Region, China; b The Department of Respiratory and Critical Care Medicine, The 924th Hospital of the Joint Logistics Support Force of the Chinese People’s Liberation Army, Guilin City, Guangxi Zhuang Autonomous Region, China; c The Department of Respiratory and Critical Care Medicine, The 924th Hospital of the Joint Logistics Support Force of the Chinese People’s Liberation Army, Guilin City, Guangxi Zhuang Autonomous Region, China.

**Keywords:** BALF, freshwater crab, misdiagnosis bronchoalveolar lavage fluid, *Paragonimus* eggs, pulmonary paragonimiasis, treatment

## Abstract

**Rationale:**

Paragonimiasis, a rare parasitic disease, often presents diagnostic challenges due to its insidious onset, multiorgan involvement, and nonspecific clinical manifestations, frequently leading to misdiagnosis. While numerous reports describe diagnostic errors in paragonimiasis management, cases involving repeated misdiagnoses across multiple tertiary hospitals over 2 years (particularly with comprehensive epidemiological evidence of freshwater crab consumption) remain exceptionally uncommon. We herein present such a noteworthy case.

**Patient concerns:**

A 45-year-old male was hospitalized 5 times across 4 tertiary centers in Guilin (January 2023–October 2024) for recurrent cough, hemoptysis, and persistent eosinophilia (lasting 2 years). Initial leukocytosis normalized, while serial chest computed tomography (CT) revealed dynamic, migratory pulmonary lesions. Extensive investigations (X-pert/T-pert, repeated bronchoscopy with bronchoalveolar lavage fluid next-generation sequencing) were nondiagnostic, leading to successive misdiagnoses of viral pneumonia, mycoplasma infection, pulmonary mycosis, tuberculosis, and suspected malignancy.

**Diagnoses:**

During the hospitalization at the 924 Hospital, a bronchoscopy was performed. Bronchoalveolar lavage fluid high-throughput sequencing revealed the presence of *Paragonimus westermani* (sequence count: 6). Upon further inquiry, the patient reported a history of capturing and consuming raw freshwater crabs during a trip to Guizhou Province in June 2022, with a self-recorded video of this activity shared on social media. Subsequent diagnostic evaluation included multiple sputum examinations for parasitic eggs. *Paragonimus* eggs were identified during the third sputum concentration test, confirming a diagnosis of paragonimiasis.

**Interventions:**

Treatment consisted of oral praziquantel (0.2 g 3 times daily for 3 days), followed by a second cycle of anthelmintic therapy after a 1-week interval.

**Outcomes:**

During the 4-month postdischarge period, the patient remained asymptomatic with complete resolution of both cough and hemoptysis. Serial laboratory monitoring demonstrated normalization of previously elevated eosinophil counts. Follow-up chest CT revealed significant radiographic improvement, including the complete disappearance of the irregular nodule in the left upper lobe and marked resolution of perilesional infiltrates surrounding the right pulmonary cavity.

**Lessons:**

In cases presenting with recurrent hemoptysis, persistent eosinophilia, and a history of raw crustacean consumption, accompanied by dynamic migratory pulmonary infiltrates on serial CT imaging, paragonimiasis should be strongly suspected despite initial diagnostic challenges.

## 1. Introduction

*Paragonimiasis* is a foodborne zoonotic disease caused by *Paragonimus westermani*. The genus *Paragonimus* encompasses a diverse array of trematode species, over 10 species of *Paragonimus* are pathogenic to humans, with *P westermani* and *P skrjabini* being the most common. The disease is insidious in onset, manifests with nonspecific and complex clinical features, and exhibits low egg detection rates, leading to frequent underdiagnosis or misdiagnosis, The misdiagnosis rate ranges from 68.75% to 88.60%.^[1]^ Although misdiagnosis and inappropriate management of *Paragonimiasis* are commonly reported, cases involving misdiagnosis by multiple tertiary hospitals, a 2-year-long well-documented clinical history and epidemiological evidence of raw freshwater crab consumption remain exceptionally rare. This case is reported as follows, expecting to provide insights for clinical practice.

## 2. Case report

**Figure 1.**
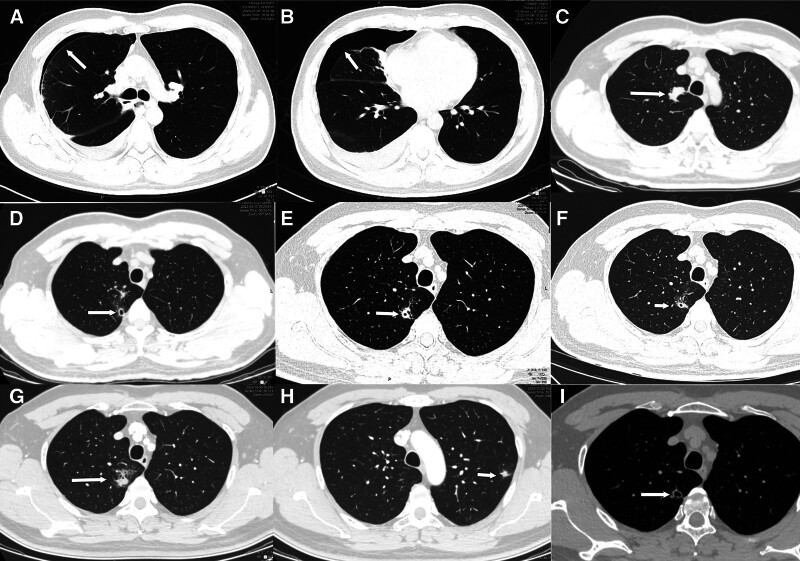
Serial chest CT imaging demonstrating dynamic pulmonary lesions: (A and B) January 8, 2023 (initial presentation), right-sided hydropneumothorax with scattered exudative opacities and peripheral infiltrative changes in the right lung; (C) June 9, 2023: lobulated irregular hyperdense lesion in the right upper lobe; (D) September 10, 2023, partial resolution and positional shift of the right upper lobe hyperdensity with cavitation; (E) May 13, 2024, cavity wall thickening and peri-cavitary exudative changes in the right upper lobe; (F) May 20, 2024, reduced cavity wall thickness in the right upper lobe; (G and H) October 30, 2024, complete resolution of the right upper lobe cavity with recurrent hyperdense lesions and new irregular nodules in the left upper lobe apicoposterior segment; (I) February 13, 2025, significant absorption of peri-cavitary exudation and resolution of left upper lobe nodules, no new lesions detected. CT = computed tomography.

A 45-year-old male was admitted to Guilin Second People’s Hospital on January 9, 2023, with a 15-day history of cough and sputum. A complete blood count (CBC) included leukocytosis (13.2 × 10⁹/L), neutrophilia (4.65 × 10⁹/L), and marked eosinophilia (6.64 × 10⁹/L). Hepatic/renal function profiles, cardiac enzymes, serum electrolyte levels, and tumor marker panels were normal. Syphilis antibody was positive (TRUST negative). Chest computed tomography (CT) revealed right-sided hydropneumothorax (30% lung compression), scattered inflammatory infiltrates in the right lung lobe, and localized lobular emphysema in the right lung lobe (Fig. 1A and B). The patient was admitted with a diagnosis of right-sided spontaneous pneumothorax and viral pneumonia. Treatment included antitussive therapy, mucolytic agents, and adjunctive therapies to enhance pulmonary circulation and tissue repair, followed by discharge after clinical improvement.

On June 9, 2023, the patient was admitted to Guilin Medical University Affiliated Hospital due to cough and sputum production for 4 days. A CBC showed leukocytosis (9.22 × 10⁹/L), marked eosinophilia (2.167 × 10⁹/L), serum immunoglobulin E (IgE) (>6000 ng/mL), erythrocyte sedimentation rate (27 mm/h). Hepatic/renal function profiles, cardiac enzymes, serum electrolyte levels, tumor marker panels, (1,3)-β-D-glucan test (G-test), *Mycoplasma pneumoniae, Chlamydia spp*., *Legionella pneumophila, Coxiella burnetii*, adenovirus, respiratory syncytial virus, influenza A/B, and parainfluenza virus were all within normal limits. The tuberculin (purified protein derivative) test was negative with 0 mm induration. Stool microscopy revealed no parasitic ova/cysts (*Enterobius vermicularis, Ascaris lumbricoides, Ancylostoma duodenale, Trichuris trichiura, Clonorchis sinensis, Strongyloides stercoralis*, and *Blastocystis hominis*). Sputum analysis demonstrated no malignant cells, fungal elements, or acid-fast bacilli. Contrast-enhanced CT showed right upper lobe patchy opacities, mild mixed-type emphysema involving both lung, and right middle lobe fibrosis (Fig. 1C). Bronchoscopy performed on June 12 revealed inflammatory changes in the trachea and subsegmental bronchi (up to the 4th order). Bronchoalveolar lavage fluid (BALF) analysis demonstrated absence of neoplastic cells, *Mycobacterium tuberculosis*, or fungal elements on direct microscopy. Liquid-based cytology identified ciliated columnar epithelial cells, macrophages, and neutrophils, with no malignant components detected. Next-generation sequencing of BALF showed no pathogenic bacteria, viruses, parasites, or *M tuberculosis* complex. Follow-up CBC on June 15 included leukocytosis (7.11 × 10⁹/L), marked eosinophilia (1.863 × 10⁹/L). The patient was diagnosed as community-acquired active pneumonia, non-severe bronchial asthma pending exclusion. The patient received anti-infective therapy, antitussives, and mucolytic agents. Symptomatic improvement was achieved prior to discharge.

On September 10, 2023, the patient was admitted to Guilin Medical University Affiliated Hospital due to intermittent cough and hemoptysis for over 1 month. A CBC showed leukocytosis (6.66 × 10⁹/L), marked eosinophilia (0.573 × 10⁹/L), and IgE (4749 ng/mL). C-reactive protein (CRP), hepatic/renal function profiles, cardiac enzymes, electrolyte levels, coagulation parameters, tumor markers, tuberculosis antibodies, and respiratory pathogen panels (*M pneumoniae, Chlamydia spp., L pneumophila, C burnetii*, adenovirus, respiratory syncytial virus, influenza A/B, parainfluenza virus) were all within normal limits. G-test and galactomannan test returned negative results. Sputum cytology and microbiological examinations demonstrated no malignant cells, *M tuberculosis*, or fungal elements. Electrocardiography indicated sinus bradycardia without acute ischemic changes. Pulmonary computed tomography angiography suggested the apical segment of the right upper lobe infection (tuberculosis not excluded) and mild mixed-type emphysema involving both lung (Fig. 1D). A repeat bronchoscopy on September 14 revealed inflammatory changes in the trachea and subsegmental bronchi (up to the 4th order) with minor bleeding at the right upper lobe orifice. BALF analysis via 198-pathogen targeted sequencing detected *M pneumoniae* (sequence count: 41 287) and a positive G-test at 989.33 pg/mL (threshold: >100 pg/mL), though no fungal, bacterial, or mycobacterial pathogens were identified. Diagnoses included non-severe community-acquired pneumonia, mycoplasma infection, and airway hyperreactivity. The patient received empirical antimicrobials, antifungals, antitussives, and hemostatic agents, yielding partial symptom resolution but persistent hemoptysis (dark-red sputum).

**Figure 2.**
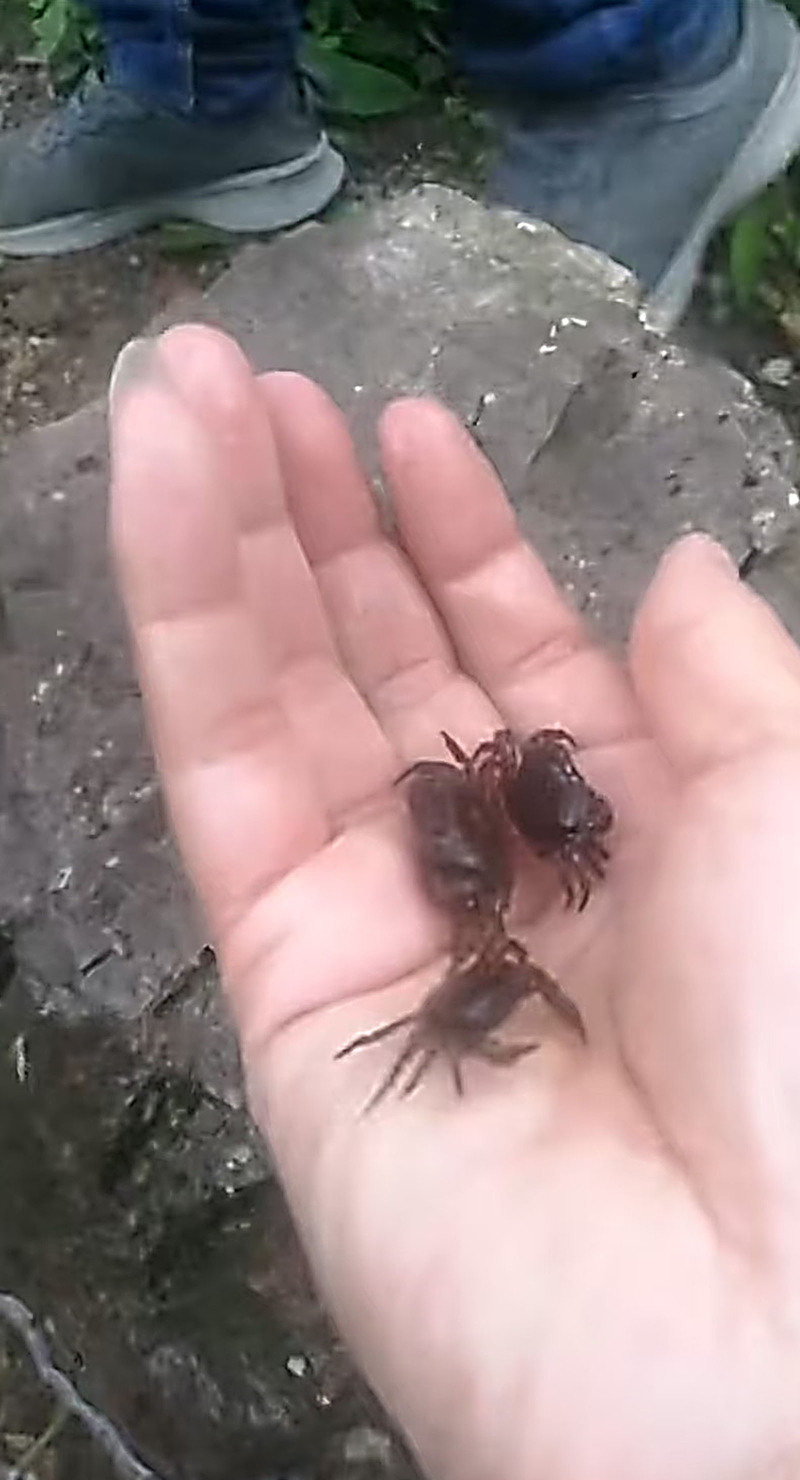
Photograph documenting the patient capturing stream crabs during a freshwater excursion.

**Figure 3.**
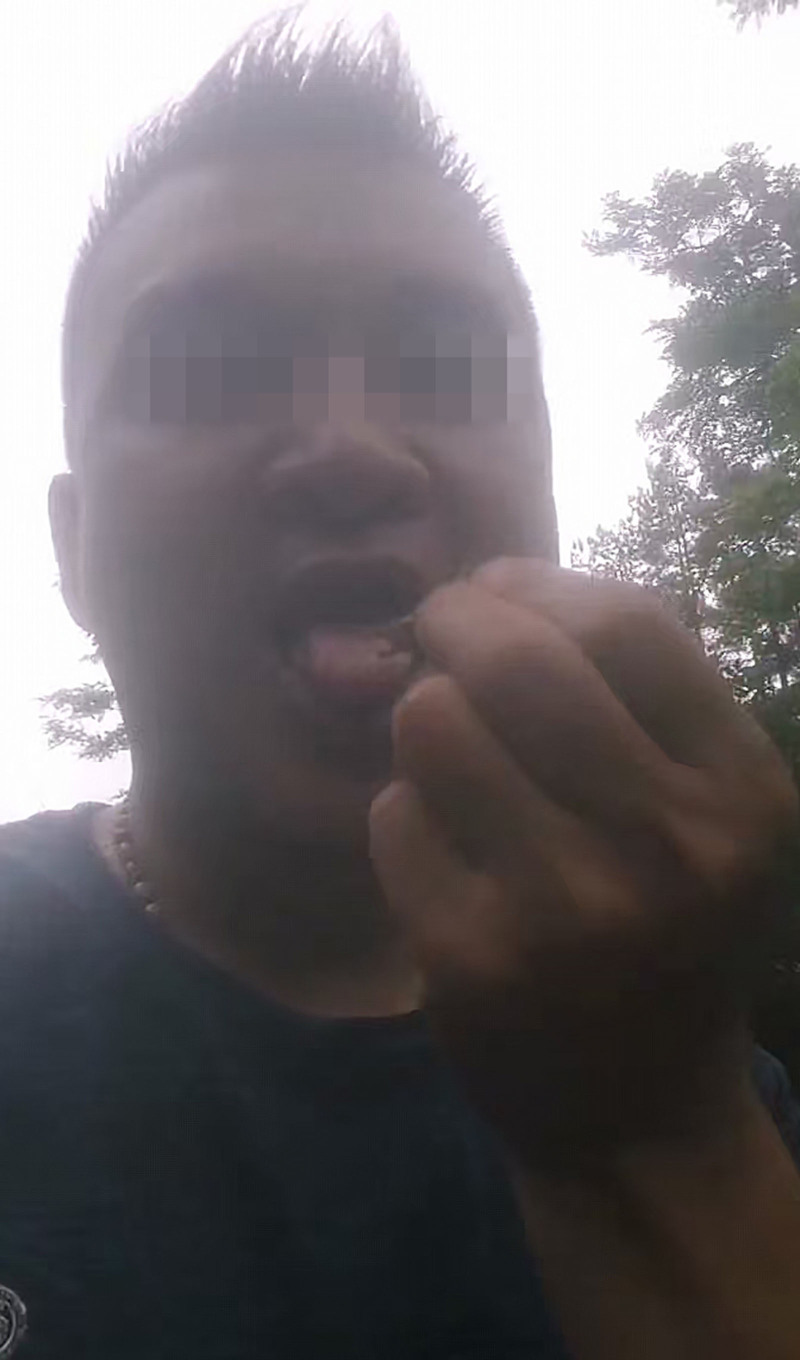
Photographic evidence of the patient consuming raw stream crabs.

**Figure 4.**
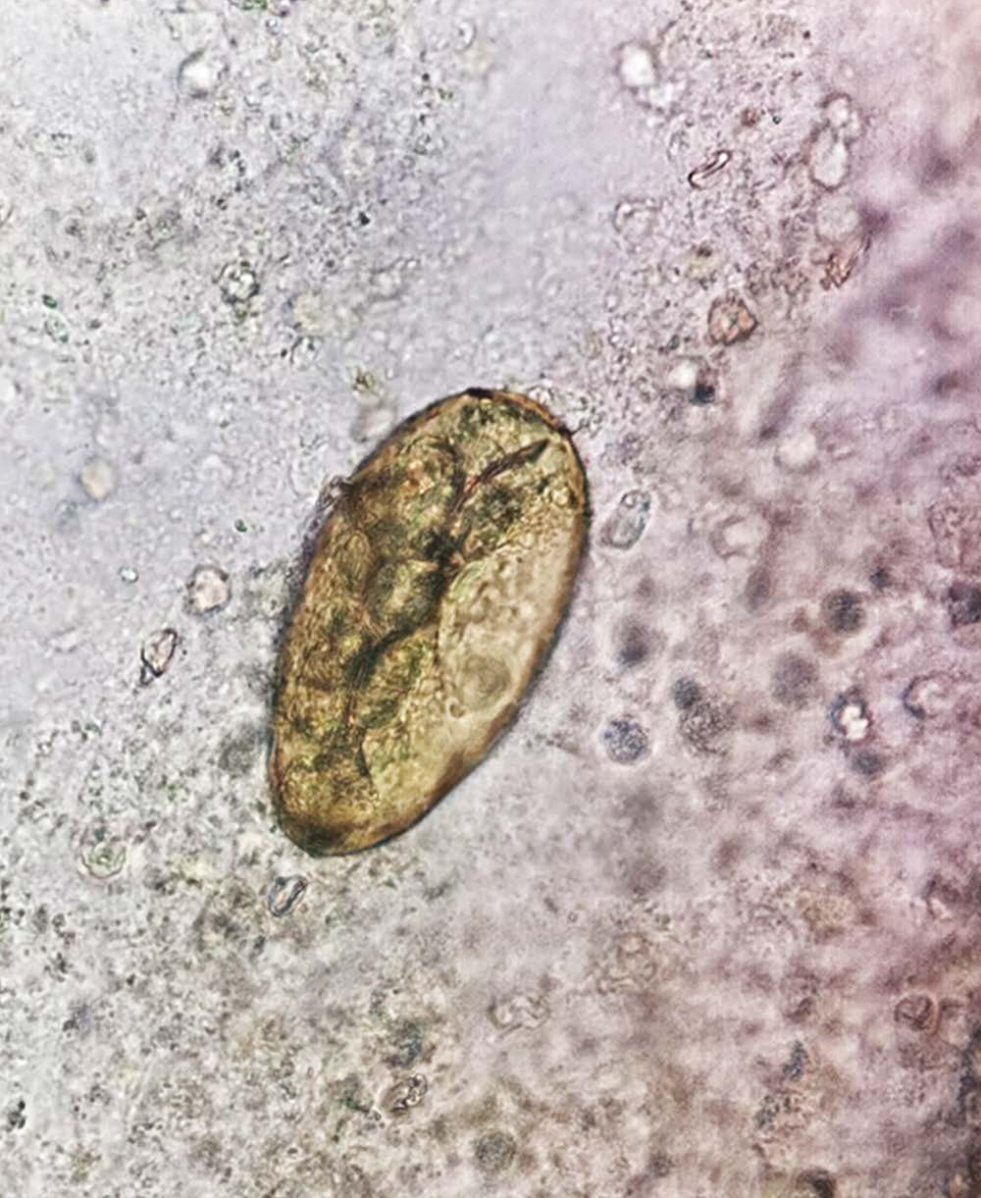
Microscopic identification of *Paragonimus* eggs in sputum, with the operculum clearly visible.

On May 13, 2024, the patient presented to Guilin Third People’s Hospital for medical evaluation due to persistent intermittent bloody sputum. Chest CT at the outpatient clinic revealed infectious lesions in the right upper lobe with cavitation suggestive of tuberculosis and localized emphysema in the right upper lobe (Fig. 1E). Hospitalized on May 20, a CBC showed leukocytosis (5.84 × 10⁹/L), eosinophil (0.77 × 10⁹/L), with normal hepatic/renal function profiles, cardiac enzymes, electrolyte levels, and pretransfusion tests. Testing for *M pneumoniae, Chlamydia* DNA, and *Bordetella pertussis* nucleic acid returned negative results. Repeated sputum examinations revealed no fungal, bacterial, tuberculous, or neoplastic cells. Stool analysis demonstrated no ova of *A lumbricoides, A duodenale*, or *T trichiura*. *M tuberculosis* DNA testing showed no abnormalities, with both IgG and IgM antituberculosis antibodies testing negative. T-cell-based tuberculosis infection assays were negative, and the X-pert MTB/RIF also yielded negative results. Bronchoscopy on May 24 indicated chronic bronchitis. Six BALF smears and bronchial brushings showed no evidence of tuberculosis, fungi, or malignant cells. Chest CT revealed infectious lesions in the right upper lung, suggestive of tuberculosis with cavitation, and localized emphysema in the right upper lung (Fig. 1F). However, hemoptysis persisted despite treatment. In October 2024, the patient was admitted to PLA 924 hospital with recurrent cough and hemoptysis for nearly 1 year, no night sweats or afternoon fevers, and no history of raw fish consumption. Physical examination revealed coarse breath sounds without rales. A CBC showed leukocytosis (6.80 × 10⁹/L), eosinophil (0.38 × 10⁹/L), erythrocyte sedimentation rate (26 mm/h), and antinuclear antibody titer (1:100), while other autoimmune antibodies, procalcitonin, CRP, hepatic/renal function profiles, cardiac enzymes, electrolyte levels, coagulation profiles, tumor markers, and routine urinalysis/stool tests were normal. Testing for *Influenza A/B virus, Respiratory syncytial virus* (*RSV*) *RNA, M pneumoniae DNA*, and *Chlamydophila pneumoniae DNA* returned negative results. Sputum smear examination revealed no acid-fast bacilli. Electrocardiogram demonstrated sinus rhythm with normal waveforms. Chest CT revealed shadows in the right upper lobe apical segment and left upper lobe apicoposterior segment suggestive of tuberculosis, bilateral upper lobe emphysema, and fibrotic streaks in the right middle lobe medial segment (Fig. 1G and H). Bronchoscopy on November 1 identified hemorrhage in the right upper lobe apical segment. BALF smears showed no bacteria, fungal spores, or acid-fast bacilli, but metagenomic sequencing detected *Paragonimus westermani* (sequence count: 6; Fig. S1, Supplemental Digital Content, https://links.lww.com/MD/Q418). Based on the clinical findings suggesting suspected pulmonary paragonimiasis, the patient was repeatedly inquired regarding prior consumption of raw or undercooked crayfish or crabs to assess potential exposure history, the patient recalled consuming raw freshwater crabs during a 2022 trip to Guizhou with a self-recorded video of this activity shared on social media (Figs. 2 and 3). Subsequent third sputum concentration test confirmed *Paragonimus* eggs (Fig. 4), leading to a diagnosis of paragonimiasis. On November 5, 2024, the patient received oral praziquantel tablets at 0.2 g 3 times daily for 3 consecutive days, followed by a second cycle of antiparasitic therapy after a 1-week interval. Following treatment, the patient was discharged with resolution of recurrent hemoptysis. During the 4-month post-discharge follow-up period, no recurrence of cough or hemoptysis was observed. Repeat evaluation on February 13, 2025, demonstrated normalization of eosinophil count and chest CT found no new lesions detected, complete resolution of the irregular nodule in the left upper lobe, and significant absorption of perilesional exudative changes surrounding the right pulmonary cavity, the patient’s entire disease course is as outlined in timeline (Table S1, Supplemental Digital Content, https://links.lww.com/MD/Q417).

## 3. Discussion

The *Paragonimus* species are hermaphroditic trematodes characterized by an ovoid morphology, with a tegument covered by smooth muscle and cuticular spines. The anterior end features an oral sucker, while a ventral sucker is located mid-body. The digestive system comprises a mouth, pharynx, esophagus, and paired intestinal branches. The reproductive system includes a lobulated ovary and uterus positioned posterior to the ventral sucker, with 2 branched testes occupying the posterior third of the body. Morphological differentiation relies on body dimensions, ovarian/testicular lobulation patterns, and spine distribution. The operculated eggs are elliptical, golden-brown, with a prominent operculum, containing a single ovum and multiple yolk cells. In this case, sputum analysis confirmed the presence of *Paragonimus* eggs, with the operculum distinctly visible.

Human infection with *Paragonimus* occurs through consumption of raw or undercooked freshwater crabs, crayfish (*Cambaroides spp.*), and other aquatic products contaminated with metacercariae. Ninety percent of the cases occurred in Asia.^[2]^ Japan reports the most commonly. Paragonimus infection prevalence is influenced by regional cultural differences and food preparation practices, such as alcohol- or soy sauce-marinated crabs; Matsuoka et al reported a case of paragonimiasis in a patient and family members due to frequent consumption of undercooked crabs^[3]^; Kashida et al reported a case of cerebral paragonimiasis in an elderly woman presenting with severe headache, attributed to frequent consumption of *Eriocheir japonicus* (Japanese mitten crab)^[4]^; Nakagawa et al described an outbreak of *Paragonimus westermani* infection among Cambodian technical interns in Japan who purchased *Eriocheir japonica* crabs from a local grocery store near their training site^[5]^; additionally, reports indicate the average incubation period of *Paragonimus westermani* is 6 months, consistent with this case where symptoms manifested 6 months after the patient consumed raw stream crabs from June 2022 to January 2023.

Paragonimiasis lacks pathognomonic clinical manifestations and is categorized into 4 types based on the anatomical involvement: thoracopulmonary (fever, cough, productive sputum, chest pain, and hemoptysis), abdominal (abdominal pain, palpable masses), neurologic (headache, vomiting, and seizures), and cutaneous (migratory subcutaneous nodules, predominantly in the lower abdomen, and thighs). Physical examination often reveals nonspecific findings. Laboratory tests may reveal elevated white blood cell counts and CRP levels, which may return to baseline levels over time. Notably, in this case, the patient’s eosinophil levels remained above the normal range for over 1 year (Fig. S2, Supplemental Digital Content, https://links.lww.com/MD/Q418). In patients with paragonimiasis, peripheral blood eosinophilia is commonly elevated, but the degree of elevation does not correlate with infection severity, and eosinophil levels may remain unremarkable in advanced stages. Elevated eosinophils lack diagnostic specificity for paragonimiasis, as allergic diseases and hematologic malignancies can also induce eosinophilia. A retrospective analysis of 30 paragonimiasis cases reported eosinophil elevation in both blood and pleural fluid in all patients.^[6]^ Based on in vitro experiments and human data, reviews have also reported the dual roles of eosinophils in parasitic infections: protective immunity or pathological effects.^[7]^ Although parasitic infections represent one of the most common etiologies of blood eosinophilia, they are frequently overlooked in clinical practice.^[8]^ Chest CT findings in paragonimiasis include pneumothorax with effusion, nodules, cavities, hemorrhagic foci, “tunnel” signs, and “halo” signs. Intracranial hemorrhagic lesions and extensive “finger-like” edema are evident on cranial CT and magnetic resonance imaging. Abdominal CT may reveal ascites and abdominal wall nodules. Serpentine deformities of the brain, lungs, liver, and spleen and “tunnel” signs are characteristic.^[7]^ Due to the heterogeneity and nonspecificity of CT manifestations, paragonimiasis is frequently misdiagnosed as tuberculosis, lung cancer, or metastatic disease.

The definitive diagnosis of paragonimiasis primarily relies on the detection of *Paragonimus* eggs. Diagnosis is established based on epidemiological history, clinical manifestations, and laboratory investigations. Current laboratory diagnostic methods for paragonimiasis include 3 main tests: the enzyme-linked immunosorbent assay (ELISA), the dot immunogold filtration assay, and the paragonimiasis antigen intradermal test.^[9]^ Each test has distinct advantages and limitations. Paragonimiasis antigen intradermal test is characterized by its simple, rapid, and cost-effective procedure, making it suitable for clinical promotion. ELISA has been reported to demonstrate up to 100% positivity rate, while exhibiting cross-reactivity with multiple parasite species^[10]^ and enables quantitative detection of *Paragonimus*-specific antibody titers and is applicable for therapeutic efficacy evaluation. *Paragonimus* eggs are typically detected in stool, sputum, or BALF. However, studies report a low egg detection rate of only 11.7% in sputum or stool.^[11]^ Cases confirmed by identifying eggs in BALF have been reported.^[12]^ In this case, *Paragonimus* eggs were identified in BALF and detected in the third sputum examination, demonstrating that multiple sputum examinations can improve egg detection rates. The WHO recommends triclabendazole and praziquantel as first-line treatments for paragonimiasis. Zheng et al conducted a retrospective analysis of 72 paragonimiasis cases treated with praziquantel, demonstrating 100% efficacy with no significant adverse effects reported.^[13]^

The misdiagnosis of this case across multiple hospitals can be attributed to the following factors based on literature and clinical practice analysis. Paragonimiasis is a rare disease, laboratory departments seldom perform diagnostic tests such as the paragonimiasis intradermal test, double immunodiffusion test, immunoenzymatic assays, or ELISA, while the detection rate of *Paragonimus* eggs in stool examinations remains low, even when clinicians suspect the disease, diagnostic confidence is compromised. The patient presented primarily with hemoptysis, and chest CT revealed lesions predominantly in the right upper lung lobe, a predilection site for tuberculosis, leading clinicians to prioritize tuberculosis as the initial diagnosis. Consequently, extensive investigations failed to confirm the etiology. Marked eosinophilia was elevated notably. During the second hospitalization, the elevation of marked eosinophilia prompted evaluations for *Paragonimus* eggs in stool, sputum, and BALF, yet none were identified. The elevated eosinophil count and IgE levels instead raised suspicion for asthma. Clinicians demonstrated inadequate elicitation of epidemiological history across 3 hospitals and 4 admissions, with no documentation of raw freshwater crab consumption or comprehensive inquiry into potential exposures such as alcohol-marinated crabs, undercooked shrimp/crab, snails, semi-raw fish, or crustaceans. Literature reports indicate that drinking stream water, using shared cutting boards for raw and cooked foods, and even venison consumption may transmit Paragonimus infection, with documented cases of viable *Paragonimus* westermani metacercariae detected in deer meat.^[14]^ Clinicians failed to recognize critical longitudinal data. The patient’s medical records from 5 admissions across 4 hospitals revealed persistent eosinophilia for approximately 1.5 years, recurrent bloody sputum, and dynamic CT findings showing migratory pulmonary lesions. These features with relative specificity were not comprehensively analyzed, underscoring the necessity of continuity in diagnostic evaluation.

## 4. Conclusion

The clinical features of this case suggest that when faced with repeated unclear diagnoses, recurrent hemoptysis, persistent eosinophilia, a history of consuming raw shrimp/crab, and multiple chest CT scans demonstrating dynamic and migratory lesions, paragonimiasis should be highly suspected. In this case, *Paragonimus* eggs were repeatedly identified via sputum microscopy. For thoracic-pulmonary paragonimiasis, sputum samples are the most accessible specimens, with a higher detection rate for *Paragonimus* eggs compared to pleural fluid or stool. Therefore, repeated sputum examinations are critical when paragonimiasis is suspected. Additionally, while the imaging features of paragonimiasis lack specificity, chest CT exhibited nearly all reported radiological manifestations over its prolonged course, including dynamic and migratory pulmonary lesions, which align with the migratory behavior of adult *Paragonimus* worms within lung tissue.

## Acknowledgments

We sincerely acknowledge the Department of Radiology of the People’s Liberation Army Joint Logistics Support Force No. 924 Hospital for providing imaging data, Geneseeq Technology Inc. Laboratory for contributing experimental datasets, and the patients who participated in this study.

## Author contributions

**Conceptualization:** Weilu Zhu, Junzhang Qin.

**Data curation:** Junzhang Qin, Weilu Zhu.

**Project administration:** Zhen Xiong, Junzhang Qin.

**Supervision:** Zhen Xiong.

**Validation:** Zhen Xiong, Junzhang Qin.

**Writing – original draft:** Junzhang Qin.

**Writing – review & editing:** Junzhang Qin, Weilu Zhu.

## Supplementary Material


