# Supplementary material for: High-throughput sequencing of bronchoalveolar lavage fluid confirms pulmonary paragonimiasis: A case report
Source: Medicine (Baltimore). 2025 Oct 24;104(43):e45261. doi: 10.1097/MD.0000000000045261 (PMC12558300; doi:10.1097/MD.0000000000045261)

**supplementary figure 1** NGS of BHLF


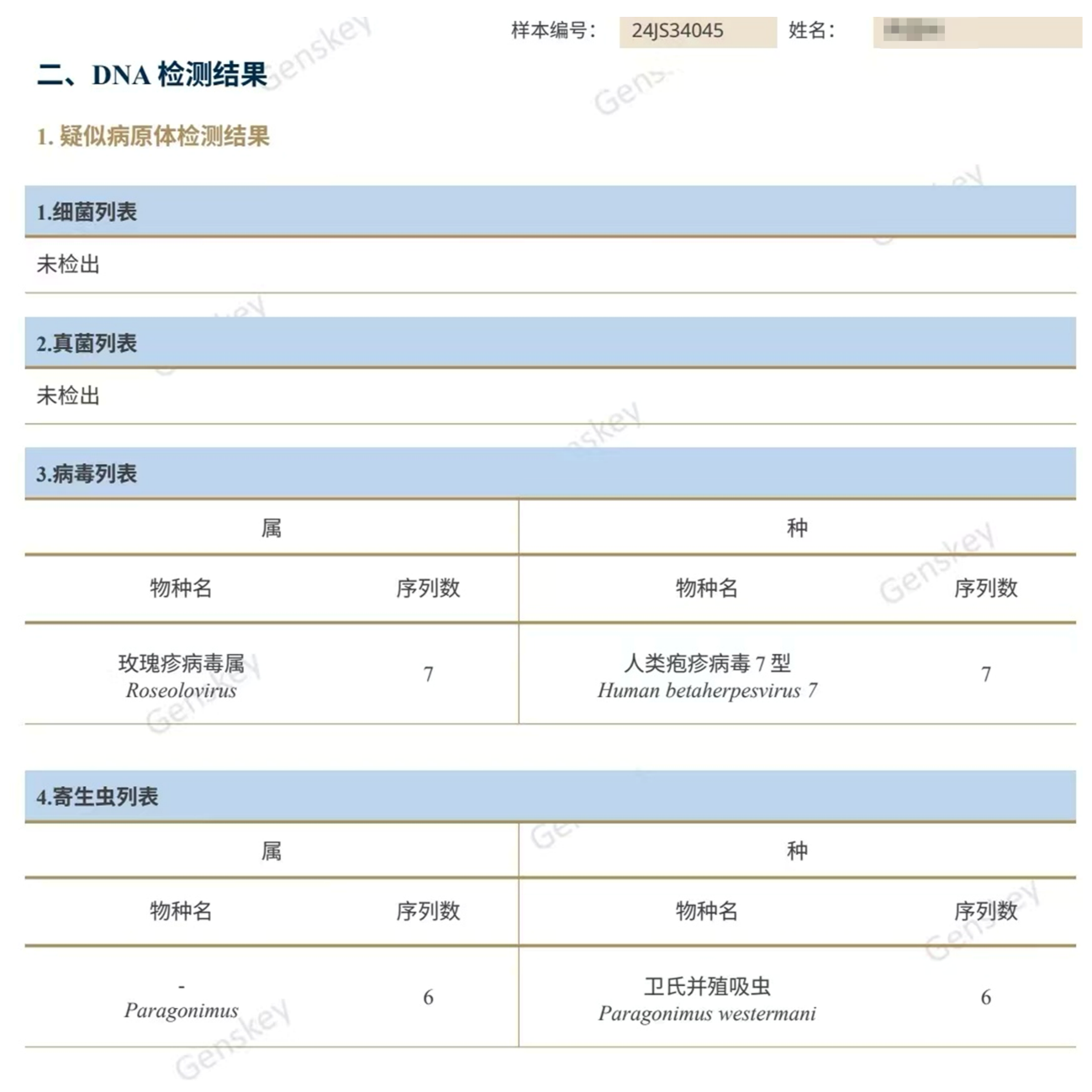


**supplementary figure 2** Longitudinal Trends in Leukocyte and Eosinophil Counts


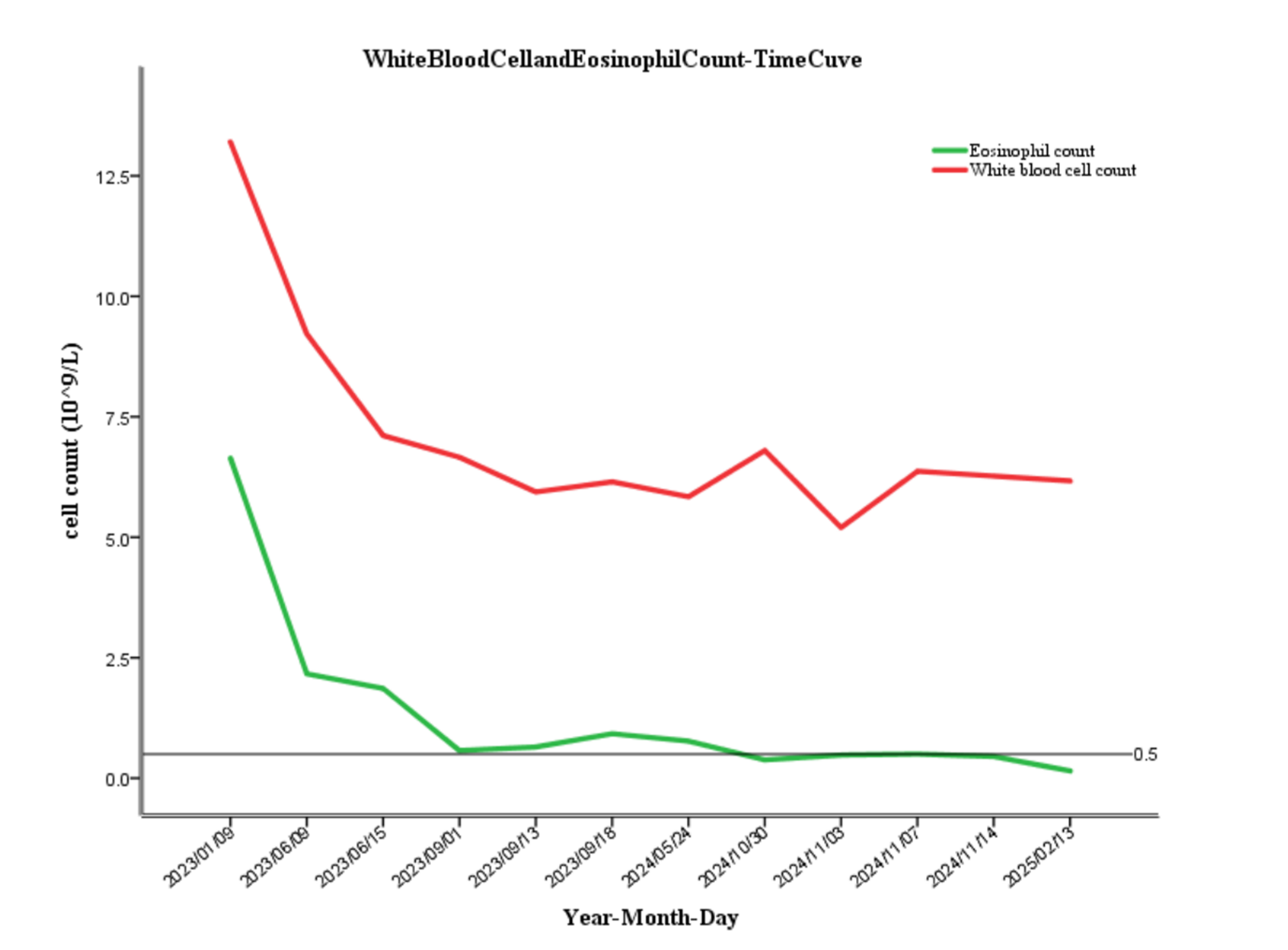

Supplement: Supplementary file 1 [file medi-104-e45261-s001.docx]
